# An investigation of foreign language writing anxiety and its reasons among pre-service EFL teachers in Pakistan

**DOI:** 10.3389/fpsyg.2022.947867

**Published:** 2023-01-04

**Authors:** Ushba Rasool, Jiancheng Qian, Muhammad Zammad Aslam

**Affiliations:** ^1^School of Foreign Languages, Zhengzhou University, Zhengzhou, Henan, China; ^2^School of Multimedia Technology and Communication, Universiti Utara Malaysia, Sintok, Malaysia

**Keywords:** foreign language anxiety, EFL, pre-service, writing anxiety, EFL teachers

## Abstract

Psychologically complicated by nature, anxiety refers to feelings of worry, fear, or apprehension. Several research studies have been devoted to exploring anxiety's effects on language skills, including writing. Since foreign language anxiety directly influences a learner's motivation and determination to learn that language, it is imperative to study the findings and reasons behind these anxious feelings. One-third of foreign language learners have been experiencing at least a moderate level of anxiety. Researchers have attempted to investigate the causes of anxiety among foreign language pre-service teachers. The present study objectifies two goals to determine the extent of writing anxiety, followed by reasons and references to the role of gender. Seventy-two pre-service teachers of the English language training department from the University of Education, Multan, Pakistan, were selected for the study using convenience sampling. Second language writing anxiety inventory (SLWAI) and second language writing anxiety reasons inventory (SLWARI) were used to collect data, and semi-structured interviews were taken with students. The findings presented no difference in anxiety levels between genders, whereas cognitive anxiety type was distinctive in results. Most of the participants experienced high and medium levels of anxiety.

## Introduction

Whether in native language (L1) or a foreign language (L2), writing is always considered a cognitively complex and demanding task as a skill, since, in Myles (2002) words, being proficient in the skill area requires conscious effort and a great deal of practice in composing, developing, and analyzing ideas. Bloom (1985) describes writing anxiety as “a term for one or a combination of feelings, attitudes, or behaviors that interfere with a person's capacity to begin, work on, or complete a particular writing job that he or she is cognitively capable of completing”. As underlined by Pajares and Johnson (1995), these unpleasant emotions and anxiety have a crippling effect on the ability of learners to write, resulting in avoidance of writing and writing classes, which subsequently leads to poor performance. Learners face difficulty in writing skills in English as a foreign language; resultantly, they experience writing anxiety (Aydin and Ciǧerci, 2020). To date, language anxiety research has aimed to uncover the reasons for writing anxiety in many aspects of writing skills. The issue at hand has been far from satisfactory from a foreign language learning perspective, particularly in the setting of English as foreign/second language (EFL/ESL) teachers (as learners/students), whose future classroom practices may influence their students' writing skills (Atay and Kurt, 2006) which ultimately lead them to write anxiety. Thus, the present study aims to determine the extent of writing anxiety experienced by ESL teachers (who attended the writing classes during their pre-service training), taking into consideration participant-related factors like gender and anxiety types and also aims to determine the possible factors that cause writing anxiety among pre-service teachers. Moreover, the research gap would be the study on the EFL teachers that have not been conducted on the said geographical and academic population. For instance, the teachers' writing skills may not be convincing as the research findings reported high and medium anxiety levels. Therefore, not only the students experience writing anxiety but also the teachers.

Language anxiety is defined as “a distinct complex of self-perceptions, beliefs, feelings, and behaviors related to classroom language learning arising from the uniqueness of the language learning process” (Horwitz et al., 1986, p. 128). Earlier to Sarason (1980) and Horwitz et al. (1986) agree that anxiety refers to a threat that is not delineated. That may further become a vague fear (see Hilgard et al., 1971). Similarly, MacIntyre (1999) defines language anxiety as nervousness, stress, worry, and emotional reaction related to language learning as a foreign/second language (L2). Therefore, language anxiety can be destructive or helpful (Alpert and Haber, 1960). Thus, writing anxiety can be caused by various factors, including a lack of knowledge of the subject matter and spelling rules and a fear of not being appreciated. Furthermore, writing ability encompasses multiple areas, such as vocabulary, grammar, phonetics, and semantics, which can cause learners to experience writing anxiety. When students write anxiously, they are more likely to make mistakes, adhering to the minutiae at the expense of the overall integrity of the text, causing a loss of authenticity in the narrative (Tayşi and Taşkin, 2018). Anxiety above the ideal level harms academic achievement (Guy and Gardner, 1985; Ehrman and Oxford, 1995; Oxford and Ehrman, 1995; MacIntyre et al., 1997). In other words, expecting learners to be concerned about various issues is acceptable. Anxiety has frequently been identified as an emotional reaction while learning any foreign language (Dewaele and MacIntyre, 2014; Boudreau et al., 2018; Oteir and Al-Otaibi, 2019; Dewaele et al., 2022). The study aimed to determine the writing anxiety level experienced by teachers and search for the difference in the levels concerning gender and the possible reasons for teachers' anxiety toward writing skills.

## Literature review

Learning a foreign language brings tremendous challenges in diverse linguistic areas and strategic, rhetorical, and cultural obstacles (Namaziandost et al., 2019). It leads a learner to experience uncomfortable or sometimes stressed feelings about the whole learning process (Jugo, 2020). Anxiety is “a feeling of wanting to do something that may happen or may have happened so that you think about it all the time or is a feeling of wanting to do something very much, but being very worried that you will not succeed” (Summers, 2007, p. 58). Foreign or second language (L2) learning always brings some mixed behavioral and linguistic concerns, which cause hindrance in performing writing tasks well (Al-Sawalha and Chow, 2012).

### Gender as a variable affecting writing anxiety

The results of an inquiry on the effect of gender on the anxiety levels of participants revealed no significant correlation between the two variables. In other words, the anxiety levels of male and female students are not related. Male students were more anxious than their female counterparts, although the difference was not statistically significant. However, an entire literature assessment on gender differences does not allow us to draw definitive conclusions. Several research appeared that unequivocally demonstrated the presence of gender differences, in contrast to others that found no correlation and concluded that gender plays no role. For example, while Shawish and Atea (2010) and Shang (2013) found no gender effect on students' writing apprehension level in favor of any group, Rodriguez et al. (2009) study found significant gender effects indicating significantly higher levels of general foreign language anxiety and writing anxiety among females. Cheng (2002) explored gender differences in skilled-specific foreign language anxiety between male and female participants. The present study added to inconclusive findings about the gender issue.

### Dimensions of writing anxiety

Many aspects of L2 learning can cause writing anxiety, such as cognitive factors and linguistic features, such as limited vocabulary, content, and structure (Daud et al., 2016). Cheng (2004) separated writing anxiety into three-dimension as Cognitive, Somatic anxiety, and avoidance behavior. Cognitive anxiety refers to pessimistic perceptions about writing and feeling of negative evaluation. In contrast, somatic anxiety refers to physical actions such as trembling, sweating and uncomfortable breathing. Avoidance behavior displays activities related to avoiding situations when someone has to write. Xu et al. (2020) discovered cognitive anxiety to be the most severe, in contrast to Atay and Kurt (2006) in Turkey and Arindra and Ardi (2020) in Indonesia, whose participant's experienced somatic anxiety-associated with physiological arousal as their dominating form. It indicates that when completing a writing assignment, participants frequently worried excessively about what other people might think and had low standards for their writing (Cheng, 2004). Similar results were obtained in Korea (An et al., 2022) with a sample of university students with a variety of competence levels and in Jordan with first-year medical students who had intermediate English proficiency (Rabadi and Rabadi, 2020). Cognitive anxiety was discovered to be the predominant type in both situations.

In contrast to the other two dimensions, Jeon's trial with learner-centered EFL writing instruction was unable to reduce participants' Somatic anxiety significantly. He attributed the predominance of cognitive anxiety among his participants to the Korean Confucian culture, which held that it was always important to be aware of other people's viewpoints to avoid facing awkward situations. This justification might also be used to explain our Chinese participation because Hong Kong is a society with a strong Confucian background. Poor writing performance was discovered to be caused by cognitive anxiety (Cheng, 2004); this anxiety was frequently triggered by writing for assessments or tests (Arindra and Ardi, 2020).

The negative associations between anxiety and writing performance have already been shown (Cheng et al., 1999; Hassan, 2001), and it has been asserted that anxiety leads to “writer's block” (Leki, 1999, p. 65) and avoidance behavior. As a result, the work of nervous students does not reflect the effort expended. According to Daly (1975), their results are lower on standardized writing tests, and their compositions are of lesser quality and less competent in the grammatical organization. Similarly, Daly and Miller (1975) emphasized that individuals with high anxiety and fear of unfavorable evaluation do not attend classes where writing is necessary and display negative attitudes toward writing. Thus, elucidating the underlying causes may provide a deeper understanding of potential remedies to boost the self-confidence and competence of students.

### Factors causing writing anxiety

Many researchers found that one of the primary reasons causing writing anxiety is a lack of writing practice throughout an academic career (Rabadi and Rabadi, 2020). Effective aspects such as aversion to writing, fear of criticism, and anxiety of being judged were also reported among anxious writers (Cheng et al., 1999; Vanhille et al., 2017; Rabadi and Rabadi, 2020). Meanwhile, individual differences such as age, gender, and socioeconomic background have been linked to various writing anxiety levels (Huwari and Abd Aziz, 2011). Contextual variables related to teachers, instructional practices, and classmates, such as discouraging or strict teaching styles, disinterested writing themes and unfamiliar formats, and blatantly negative or inadequate teacher feedback may also contribute to writing anxiety (Liu and Ni, 2015). Finally, individual factors are likely to interact with contextual factors and the learning environment (e.g., family and school). Writing anxiety can lead to a discouraging attitude toward writing and low expectations and confidence in one's work. Hassan (2001) looked at EFL Egyptian learners. He discovered that those with high writing anxiety regarded writing as unrewarding and distressing. Writing anxiety has been shown to harm EFL learners' writing processes, including behavioral symptoms such as avoidance, reluctance, and procrastination, according to MacIntyre and Gardner (1994) and Ada et al. (2004). Onwuegbuzie et al. (1999), Kitano (2001), Erkan and Saban (2011), Liu and Ni (2015), and Sabti et al. (2019) have discovered a negative link in studying the relationship between writing anxiety and performance. The work of anxious authors is of worse quality and contains more errors; it is less developed, shorter, and syntactically unfinished. The result of writers with low anxiety is of higher quality, have fewer faults, and contain more paragraphs and words. Apprehension of grammar was discovered as a sub-construct of writing anxiety by Sanders-Reio et al. (2014). It was also linked to inferior writing performance. Researchers such as Erdogan (2017), Abdullah et al. (2018), Arindra and Ardi (2020); An et al. (2022) have experimented with different teaching and learning methods to reduce writing anxiety among EFL learners. They developed an evaluation rubric for writing feedback *via* a computer-aided learning system and a more learner-centered collaborative writing lesson design. Compared to the extensive study of learners' writing anxiety above, there are few studies of L2/FL teachers' writing anxiety and how it affects their writing teaching methodology.

### Studies conducted on teachers' writing anxiety

Several studies have indicated that students' writing anxiety that connected with instructors' feedback practice (e.g., Kurt and Atay, 2007; Di Loreto and McDonough, 2013; Tsao et al., 2017), highlighting the need for future studies exploring teachers' writing anxiety and its influence on their written feedback. Atay and Kurt (2006) surveyed Turkish-English teachers and found that most suffered from moderate to high writing anxiety levels, and the somatic type of anxiety was found to be the most profound. The participants who seemed anxious reported that the anxiety happened due to product-oriented writing lessons having bad previous writing experiences. Similarly, Zerey (2013) studied Turkish native pre-service EFL teachers and found that most ELT students generally experience high or average writing anxiety toward writing tasks. Moreover, participant-related variables such as gender and high school type have no significant effect on students' total writing anxiety scores. Factors other than teachers' pedagogical practices and feedback preferences play a role in students' anxiety when asked to write in L2. Erdogan (2017) experimented with co-writing activities to assess the effectiveness of elementary school teachers in reducing writing anxiety and found that interventions were effective. Kurt and Atay (2007) investigated the relationship between the writing anxiety of future Turkish teachers and the type of feedback they received. Participants who received peer feedback were much less anxious than participants who received feedback from their teachers. Although S/FL teachers are usually advanced learners highly proficient in the language they teach, therefore, are less likely to suffer from writing anxiety, existing studies (e.g., Daly et al., 1988; Zerey, 2013) found writing apprehension among pre- and in-service teachers in both L1 and S/FL settings. However, most research on the relationship between instructors' writing anxiety and writing instruction was undertaken in L1 environments throughout the 1980s. Bizzaro and Toler (1986) observed that nervous writing instructors tended to avoid conferences with students about their composition and prevent their students from making discoveries in their writings, which was detrimental to students' writing ability and desire to write. Daly et al. (1988) discovered that instructors with high anxiety levels tended to emphasize mechanical structures, whereas teachers with low anxiety levels emphasized students' creativity. The present research might influence teacher education so that prompt preventive actions and assistance could be accessible. Such an inquiry might also be helpful theoretically for enhancing knowledge of the process of writing anxiety and its far-reaching effects on students through S/FL instructors.

### Research questions

The present study aims to answer the following questions.

1. To what degree do pre-service teachers experience writing anxiety?
2. Does gender difference influence the writing anxiety scores of pre-service teachers?
3. What reasons instigate pre-service teachers' writing anxiety?

## Methodology

The study used a mixed-method approach to collect and analyze quantitative and qualitative data in a sequence. The quantitative data was collected through two writing anxiety questionnaires, and qualitative data were collected through semi-structured interviews with participants. Creswell and Clark (2018) stated that quantitative data provides a comprehensive explanation and general detail of a research problem. At the same time, qualitative data describes the rationale and reasons for observational differences in results.

### Instruments

The current study has employed two writing anxiety questionnaires. The first tool is Cheng's Second Language Writing Anxiety Inventory (SLWAI) (2004). The current study's objectives are to find the levels of anxiety and its categories in pre-service ESL teachers. Moreover, Cheng (2004) based scale items on L2 anxiety reports and suitable anxiety scales, selecting cognitive, somatic/physiological, and behavioral anxiety components. SLWAI possesses reliability with.91 Cronbach Alpha reported (Cheng, 2004). This questionnaire assesses to what extent learners experience writing anxiety in L2 writing. It is comprised of 21 questions with five Likert-scale options ranging from 1 (strongly disagree), 2 (disagree), 3 (neutral), 4 (agree), and 5 (strongly agree). The questionnaire was divided into three categories,

- Cognitive anxiety was assessed by eight items (1, 3, 6, 8, 13, 16, 19, 20).
- Somatic anxiety refers to seven items (2, 5, 7, 10, 12, 14, 18).
- Avoidance behavior consisted of six items (4, 9, 11, 15, 17, 21).

The second instrument is the Second Language Writing Anxiety Reasons Inventory (SLWARI) by Kara (2013) with Cronbach Alpha coefficient 0.91 with 0.66 construct validity. The scale describes students' attitudes and feelings about writing anxiety. It explains different reasons that cause writing anxiety among students while L2 writing. The third research tool was a semi-structured interview, as interviews in individual differences studies like anxiety play a significant role while bringing forth deeper details of the research problems (Price, 1991).

### Participants

The study was conducted on 72 students (37 male and 35 female) of prep-classes in the English language teachers training department at Education University (Multan Campus). The most significant reason for choosing the participants from the said university is that it is the only university that offers this course in the whole South Punjab region of Pakistan's province Punjab. Moreover, the first researcher had already completed a pre-service course offered by a regional public sector institution, which later discontinued this program. All participants were aged 20–24 years except four male participants ranging from 25 to 29 and had been learning English for 15–18 years. Using convenience sampling, as Cohen et al. (2007) suggested, the present research selected participants who attended the same training level at the university.

### Procedure

The study was administered in the last week of the fall semester and conducted in May-August 2021. The course is offered to be completed in three semesters (4 months each). The time and second semester were selected because participants have already experienced different writing tasks, such as comparing/contrasting and describing cause and effects and explaining merits and demerits in their first semester, January-April 2021. The last semester, September-October 2021, consisted of particle and fieldwork. After that, the first step was to get the primary information section filled in as a first language, age, gender, and years of studying English. After the basic information, participants were directed to fill out SLWAI to determine the writing anxiety they felt while doing their writing tasks. The next step was to conduct a second inventory of SLWARI to reach out to students' subjective perceptions about possible reasons causing negative feelings of anxiety. Factor analysis was conducted to determine the scale's construct validity, which resulted in good extractions. The Cronbach's Alpha internal consistency coefficient was used to calculate the scale's reliability, which was finalized after factor analysis. In addition, the scale analysis was made using SPSS 20.0 with Cronbach Alpha.90 for SLWAI and with Cronbach Alpha.95 for SLWARI. A third research tool, interviews, was conducted a week later than the first two inventories. The researcher approached more than 45 participants in total number. However, only 20 participants (13 males and seven females) volunteered to share their views in the interview about writing anxiety and the reasons those cause anxious feelings. The interviews lasted from 8 to 10 min for each participant.

### Process of quantitative data analysis

The current research study employed mixed methods to obtain more reliable results through qualitative and quantitative data. The data collected through SLWAI were processed, and the participant's total questionnaire score was summed up. The score was divided into three levels: high, average, and low anxiety. Participants had high, average, and low anxiety. The data was also processed to compare male and female participants' anxiety scores to determine if gender affects anxiety levels. According to question types as cognitive, somatic, and avoidance behavior, three anxiety categories were assessed to explore which category learners feel more than others. All participants' responses were statistically processed by statistical package for social sciences (SPSS) version 23. The second inventory SLWARI scores were also computerized through SPSS descriptive analysis to display frequencies and percentages. The mean score of all questions showed the tendency behind writing anxiety. Twenty participants and qualitative data were conducted; the third research tool, interviews, were analyzed using the content analysis technique. According to Patton (2002), this technique has been used to transform data into findings. This way, primary patterns and cues in the data are labeled, categorized, and classified.

### Method for qualitative analysis

A directed qualitative content analysis (DQCA) approach was used for the qualitative paradigm adapted from Rasool et al. (2022). In this case, the unit of analysis was interview transcriptions (Graneheim et al., 2017). Based on prior research and theory (Mayring, 2000, 2014), categories were constructed relating supervisees' academic performance to technological, behavioral, and pedagogical concerns (Elo et al., 2014). Each researcher interdependently encoded the data and reviewed the difficulties of minimizing discrepancies to promote inter-coder reliability (Vaismoradi et al., 2013; Assarroudi et al., 2018). After inter-author talks, anchored samples were categorized. Final data analysis involved extracting meaning units and a categorization matrix from examined content (Mayring, 2014).

### Qualitative content analysis (QCA)

The present study used qualitative content analysis (QCA), derived from the third author's Ph.D. study, to code interview data and the first author's previous study to code interview data (Rasool et al., 2022). It assessed data from a communication standpoint (Mayring, 2000; Kibiswa, 2019). It refined and tested data analysis categories and patterns (Hsieh and Shannon, 2005; Elo and Kyngäs, 2008; Zhang and Wildemuth, 2009; Assarroudi et al., 2018) utilizing QCA (Elo and Kyngäs, 2008). Supervisors and supervisees used Directed QCA on interview text to evaluate communication and feedback processes (Holsti, 1968). The study changed Assarroudi et al. (2018)'s directed QCA as follows:

### Sample design for qualitative analysis

Researchers acquired general research skills by selecting “important informants” (Elo et al., 2014). It advised using purposive sampling to interview willing people (Coyne, 1997), focusing on transcribed interview data (Elo and Kyngäs, 2008; Assarroudi et al., 2018).

### Data collection process

The researchers created an interview guide with open-ended questions based on the study's aims and the prior research's primary categories (Hsieh and Shannon, 2005) involving interviews and transcribing each session's data (Poland, 1995; Seidman, 2013). For this purpose, 45 students/participants were approached by the same participants already involved in the quantitative study. However, 20 students agreed to interview.

### Specifying analysis unit

The organization, individual, programmer, classroom, interview, coded text, or transcript can be analyzed (Graneheim and Lundman, 2004; Assarroudi et al., 2018). The interview (transcriptions) was specified as the unit of analysis.

### Processing of qualitative data

Interview data were examined as often as suggested; participants' educational identity, place of communication, type of communication, why it happened, and when it happened (Elo and Kyngäs, 2008; Assarroudi et al., 2018). The research-related meaning was derived from immersing data (Elo and Kyngäs, 2008; Elo et al., 2014; Assarroudi et al., 2018; Kyngäs, 2020). Key analytical categories were created (Elo and Kyngäs, 2008), identifying subcategories based on the current theoretical framework's linkages to past research (Mayring, 2000, 2014). Technical, behavioral, and classroom/meeting room problems were developed objectively. Coding standards for the primary and subcategories were described (Mayring, 2014). Coding rules clearly distinguish the main categories from the subcategories' matrix, enhancing the study's credibility. Theoretical coding rules are derived from definitions, and samples were anchored to main and subcategories based on meaning units (Mayring, 2014). Finally, the data were evaluated according to the objectives and categorization matrix by examining the content, summarizing meaning units, and applying preliminary coding (Mayring, 2000, 2014). The data were organized and categorized using inductive derivation, similarity/difference, and constant comparison (Zhang and Wildemuth, 2009).

## Findings

The study's findings bring forth levels to which learners feel anxiety categorized as high, average, and low anxiety levels. The study also compares gender influence on learners' anxiety levels and mainly faces anxiety types out of three (cognitive, somatic, and avoidance behavior).

RQ1: To what degree do pre-service teachers experience writing anxiety?

### Anxiety levels

Responding to research question 1, participants were divided into three anxiety level groups based on their summed-up scores collected by SLWAI. The three anxiety levels were determined by a score of above 75 points, showing an as high level of anxiety. A score of <57 displayed a low anxiety level, whereas 57–75 indicates an average anxiety level (adapted from Zerey, 2013). The participants' responses were processed to get total scores, leading them to a group level of anxiety. Table 1 displays three groups of anxiety levels and participants' distribution accordingly.

**Table 1:** Participants' percentages and numbers according to anxiety level.

**Categories of anxiety**	**f**	**%**
High-level anxiety	34	47.2
Average level anxiety	32	44.4
Low-level anxiety	6	8.3
Sum	72	100.00

The data collected by SLWAI responses indicated that out of 72, only six participants, with 8.3%, experienced low anxiety levels, whereas 32 subjects faced average anxiety levels. The High-level anxiety was recorded as higher than average-and-low level anxiety, as mentioned by 34 respondents with 47.2%. It shows that most students experience high anxiety levels while writing in English. Only six participants felt a low level of anxiety while writing tasks. The findings support pre-service teachers' interview responses, as most were concerned about their writing anxiety for many reasons.

One-way ANOVA analysis (see Table 2) was adopted to see whether there is any role of gender to affect anxiety levels of the participants. Three anxiety levels as high, average and low among in review male and female participant showed no significant difference (*p* = 0.944, *p* = 0.500, *p* = 0.478) respectively which means pre-service teachers writing anxiety levels has no relation to gender as variable affecting anxiety levels.

**Table 2:** One-way ANOVA results based anxiety levels in comparison to gender.

		**SS**	**df**	**MS**	**F**	**Sig**.
High-level anxiety	Between groups	0.000	1	0.000	0.005	0.944
	Within groups	5.500	70	0.079		
	Total	5.500	71			
Average level anxiety	Between groups	0.464	1	0.464	0.460	0.500
	Within groups	70.647	70	1.009		
	Total	71.111	71			
Low-level anxiety	Between groups	1.168	1	1.168	0.510	0.478
	Within groups	160.332	70	2.290		
	Total	161.500	71			

QR2: Does gender difference influence the writing anxiety scores of pre-service teachers?

### Gender as an anxiety variable

To answer research question no. 2, the participants were divided into two groups (male and female) to determine if there is any gender effect on the anxiety levels of the learners. The data collected from the Second language writing anxiety inventory (SLWAI) displayed no significant difference with a 0.860 *p*-value in anxiety scores because of gender, so it can be said that gender is not a multiple variables for anxiety. Table 3 displays the independent *t*-test results applied to gender-wise data of the Second language writing anxiety inventory (SLWAI). It can be seen that there is no noticeable difference between male and female mean scores for males (M = 54.6) and females (M = 56.3).

**Table 3:** According to gender-independent *t*-test scores.

**Gender**	**N**	**Mean**	**SD**	**t**	**df**	***P***
Female	37	54.64	16.549	−0.418	70	0.860
Male	35	56.34	17.83			

### Anxiety types

According to Second Language Writing Anxiety Inventory (SLWAI) questions (see Table 4), the questionnaire was divided into three parts according to anxiety types (cognitive, somatic, avoidance, and behavior). Cognitive anxiety means a learner's anxious behavior because of fear of negative assessment or being tested, whereas somatic anxiety refers to fearful and worrying feelings which cause physical symptoms or trembling. Avoidance behavior is when students avoid writing asks and activities involving writing.

**Table 4:** One-way ANOVA results based on three anxiety types.

	**SS**	**DF**	**MS**	**F**	**Sig**.
Between groups	806.92	2	403.46	9.75	0.000
Within groups	8,813.05	213	41.37		
Total	9,619.98	215			

The One-way ANOVA test (see Table 5) was run on the scores collected by anxiety types of scores. The in-review results showed that participants are more likely to face cognitive anxiety than somatic and avoidance behavior. Cognitive anxiety was calculated with a mean score (of M = 21.08) and somatic and avoidance behavior with a mean (of M = 17.94) and (M = 16.44), respectively.

**Table 5:** Descriptive results through one way ANOVA analysis.

**Types**	**N**	**Mean**	**STD**	**SE**	**95%CI**	
					**LB**	**UB**
Cognitive	72	21.08	7.15	0.84	19.40	22.76
Somatic	72	17.94	6.88	0.81	16.32	19.56
Avoidance	72	16.44	5.04	0.59	15.25	17.63
Total	216	18.49	6.68	0.45	17.59	19.38

RQ3: What reasons instigate pre-service teachers' writing anxiety?

To answer research question 3, the data collected by the second inventory, second language writing anxiety reasons inventory (SLWARI), was analyzed to see whether there is difference of opinion among male and female participants about reasons of writing anxiety. SLWARI inventory was divided into three sections (Zerey, 2013) related to the leading causes of anxiety: learners' feelings about writing tasks and writing skills, teachers, and writing courses and books. Further interview data was analyzed in qualitative analysis to find out what difficulties and anxious feelings teachers usually face while writing.

### Analysis of second language writing anxiety reasons inventory (SLWARI)

The responses of SLWARI inventory showed no significance differences in reasons of writing anxiety among male and female teachers (Table 6). The responses of questions related to writing course for male participants (M = 15.22, SD = 5.4) and female participants (M = 15.46, SD = 6.49) seems not different. Similarly male and female participant's perceptions about teachers and their role in their writing anxiety has no relevance with gender as a variable (M = 22.03, SD = 7.52) and (M = 22.20.06, SD = 9.18) respectively.

**Table 6:** Descriptive analysis based on three anxiety categories.

	**SLWARI items**	**N**	**Mean**	**SD**	**SE**	**95%CI**	
						**LB**	**UB**
Writing course (items 1, 6, 8, 13, 30, 31)	Male	37	15.22	5.42	0.89	13.41	17.02
	Female	35	15.46	6.49	1.09	13.23	17.69
	Total	72	15.33	5.92	0.69	13.94	16.73
Teachers (items 2, 5, 7, 9, 18, 20, 25, 26)	Male	37	22.03	7.52	1.23	19.52	24.54
	Female	35	20.06	9.18	1.55	16.90	23.21
	Total	72	21.07	8.36	0.98	19.10	23.04
Writing ability (items 3, 4, 10, 11, 12, 14–17, 19, 21–24, 27–29)	Male	37	38.73	13.20	2.17	34.33	43.13
	Female	35	40.31	16.13	2.72	34.77	45.86
	Total	72	39.50	14.61	1.72	36.06	42.94

One-way ONOVA analysis of category wise responses (see Table 7) from SLWARI with no significant difference among three major aspects involved in affecting writing anxiety showed that in present study gender as variable has no discrimination. Questions about writing course (*p* = 0.864), questions about teachers (*p* = 0.322) and about writing ability (*p* = 0.649) showed no significant difference.

**Table 7:** One-way ANOVA results based on three anxiety categories.

		**SS**	**df**	**MS**	**F**	**Sig**.
Writing course	Between groups	1.044	1	1.044	0.029	0.864
	Within groups	2,490.956	70	35.585		
	Total	2,492.000	71			
Teachers	Between groups	69.794	1	69.794	0.996	0.322
	Within groups	4,902.859	70	70.041		
	Total	4,972.653	71			
Writing ability	Between groups	45.160	1	45.160	0.209	0.649
	Within groups	15,128.840	70	216.126		
	Total	15,174.000	71			

### Descriptive analysis of (SLWARI) items

The questions related to students' feelings about writing class teachers and instructors (items 2, 5, 7, 9, 18, 20, 25, 26) displayed the positive role of teachers during writing classes. Most of participants think teachers teach and understand the subject well with (M = 2.57, SD = 1.38) whereas participants also stated that teachers answered their questions about any difficulty during writing class (M = 2.47, SD = 1.36). Less participants felt their questions were not being addressed. The teacher's writing feedback question displayed some concerns, participants stated that teachers do not provide critical feedback on their writing. Moreover, less than half of the participants were satisfied with the amount and method of feedback (M = 2.88, SD = 1.37). Another concern about teachers' methodology is the speed of classroom lessons. Some participants think teachers switch to new topics faster, making it difficult to grasp the topic (M = 2.79, SD = 1.33). More than half of students find teachers interactive and exciting while teaching. Most participants liked how teachers gave examples to make them understand the topic and guide students to write better every time. The inventory questions related to learners' feelings about teachers clearly show that most learners are happy with the way teachers do their jobs and put their maximum effort into teaching in writing classes.

When students were asked about writing classes and courses (items 1, 6, 8, 13, 30, 31), they came up with some ideas. Many of the participants think their writing difficulties are because they do not have any writing course background (M = 2.29, SD = 1.36). However, participants agreed with the number of helpful examples mentioned in the course books. When asked about course books, less students feel course books are not as interesting; on the other hand, 38 students opine course books are not boring (M = 2.75,SD = 1.44). Few participants consider course book exercises less than required, so they get fewer chances to practice what they have learned (M = 2.67, SD = 1.46). Most students like to practice writing after class to perform better in the future. After that, 66.4% of participants disagreed that “irregularity in class attendance” could be a reason for failure (M = 2.57, SD = 1.43). Overall, the course book's content seems satisfactory in the writing class process.

When participants' responses about writing ability and writing skills items (3, 4, 10, 11, 12, 14, 15, 16, 17, 19, 21, 22, 23, 24, 27, 28, 29) were analyzed, Some of participants thought they could not write about any topic because of a limited range of grammatical knowledge. Still, they get ideas but do not know how to put them together and compose them into sentences (M = 2.78, SD = 1.44). Most of participants find it easy to find a topic to write about when they want to manage any writing task. Therefore, only 25% of participants dislike writing classes (M = 2.31, SD = 1.39) and 63.9% like to attend writing courses. 65.3% of participants struggle with writing tasks because of a lack of practice and regular writing habits (M = 2.40, SD = 1.33). 54.2% of subjects cannot get an idea to start writing tasks if they have to write any composition. 47.2% of participants think writing is a delicate skill, and skills writers do practical writing tasks with (M = 3.08, SD = 1.39). 50% of participants have trouble with organizing ideas. For instance, they fail to organize what they want to write linguistically correctly, combining ideas and connecting them cohesively. 61.1% of participants could not organize the concepts with each other while writing (M = 2.54, SD = 1.36). Expression is another issue raised by participants. Putting ideas from the mind into words is hard for many of the participants M = 2.67, SD = 1.36). Many students doubt their creativity because they cannot write what they want.

### Qualitative analysis

#### Semi-structured interview questions

Semi-structured interviews are a practical approach to data collecting to collect qualitative, open-ended data; probe participants' thoughts, feelings, and beliefs about a topic; and delve deeply into personal, often critical subjects (Whiting, 2008).

Five essential questions were included in the interview, and some additional questions were asked during the interview accordingly. What do you think about your writing ability?

1. Do you feel anxious when you write in English?
2. How do you feel about the teacher's methodology for teaching writing class?
3. What do you think about course books?
4. What is the most challenging obstacle you feel when you write in English?

#### Interview responses analysis concerning SLWARI

The findings attained from the interview related to the conclusions of the second inventory SLWARI. The participants' primary reasons for writing anxiety in the interview support the responses about learners' linguistic abilities and writing skills. The participants cited a lack of vocabulary, grammatical knowledge, and problems while organizing composition. The participants struggle to write in English when they think and gather ideas in their language. Many participants did not have experience practicing writing tasks in high school and had no regular habit of writing.

The following extracts demonstrate the relevance of questionnaire responses from the participants' interview responses.

Student participant = *SP*.

#### Learner's writing ability and writing anxiety

Participants shared different opinions when asked about their writing ability and anxious feelings. Most student teachers expressed that their writing ability is of intermediate level, but they still fear making technical mistakes while writing. Many participants feel anxious when writing in English because they did not have much writing practice in high school, which caused hesitation while writing. One student teacher said, “*I think my writing ability is intermediate level, but I do feel I do still make technical mistakes when I write” (SP1)*. One of the critical points raised was that learners got fewer chances to write, and mostly they used to cram the content if they had to write. The primary concern of students was a lack of writing practice during their high school studies and fewer chances of writing. Another point related to lack of writing practice was students' cramming habits. “*I always feel some hesitation because I get fewer chances to write and mostly cram the content if I have to write” (SP3)*. Some students shared that they used to cram the content for exams and assessments for writing tasks. These reasons were significant obstacles to improving their writing ability throughout their academic period.

#### Learner's feelings about writing instructors and teaching methodology

When learners were asked about teachers and their methodology, they expressed positive remarks. They opine that their teachers tried their best to explain the writing rules and gave many examples, but since English was different from their first language, sometimes it was hard to understand effortlessly. Sometimes teachers (as learners) felt bored by many grammatical rules, although the teacher tried to make the lesson interesting and include many examples while teaching. When learners were asked about teachers and their methodology, they expressed positive remarks about it as, “*Our teacher tries her level best to explain the writing rules and give many examples, but since the English language is different from my first language so, sometimes it is hard to understand easily” (SP11)*. Moreover, a participant argued, “*sometimes I feel bored by so many grammatical rules, but our teacher tries to make the lesson interesting and includes many examples while teaching” (SP19)*. Learning speed was mainly highlighted as the reason for feeling anxious because participants stated that “*all teachers (as learners) are not equally capable of keeping pace with lessons taught” (SP11)*. However, they expressed that most teachers tried to maintain balance while teaching and jumping to another lesson. They ensured all learners were on the same page and understood what was being taught. Participants generally favored their teacher's attitude and teaching methodology. They thought the number of examples and exercises teachers provided while teaching was enough and always made learning easier for them.

#### Learner's opinion about writing courses and books

The participants reported their perception of writing books and courses precisely. They think course books contain exercises and examples which are very helpful. Some shared their concern about complicated grammar rules, which are hard to understand and not functional in oral speaking. Some of the teachers (as learners) suggested that course books can be more enjoyable. Regarding course book practice exercises, they seemed optimistic about the content; however, sometimes, grammatical rules are challenging to comprehend. Participants shared their concerns about books as, “*Some of the difficult grammar rules are hard to understand and examples are equally difficult to understand in the books” (SP8) and “I think course books can be more interesting” (SP11)*. Participants also showed their concern for course books to be less boring and need to include exciting exercises.

#### General obstacles while writing in English

During the interview, participants brought forth many reasons that cause them to feel anxious whenever they have to write in English, such as,

##### Organizing ideas

Some learners shared that when the teacher gives them any topic to write about. They come up with many ideas and points, but when they have to organize them together, they struggle. They are unable to write cohesively, which troubles them.

##### Limited vocabulary

Additionally, some always feel anxiety if they have to write in English because they cannot find the right words because of their limited vocabulary. The range of vocabulary sometimes creates writer's block. They want to write but cannot execute their ideas on paper.

##### Accurate grammar

Sometimes learners keep thinking about their written production even after submissions because they are not confident that whatever they have written is grammatically correct. Moreover, they mostly think grammatical accuracy is their weakness. Many teachers (as learners) can gather ideas but cannot fully express them in writing because they know little about grammatical complexities.

##### Examination fear

Assessment of examination fear is another brought forth reason for anxiety. Participants stated that usually, during classwork, they do not feel as much anxiety as during examinations. They think that during examinations, they get anxious about being unable to perform according to their abilities, and they will not be able to put ideas together on paper.

##### Peer pressure

A compelling reason shared by teachers (as learners) was peer pressure and being judged. Some participants also shared that they feel worried and anxious because of negative teacher feedback or evaluation. They fear the embarrassment of being unable to write up to the mark and face failure in front of their peers. They are afraid of being judged if they fail to perform well.

## Discussion

The present study aimed to explore the levels and reasons of writing anxiety learners experience and gender influence on anxiety levels. The study also brought forth the participants' mostly experienced anxiety types (cognitive, somatic, and avoidance behavior). The first research questions revealed the three levels of anxiety experienced by participants: high anxiety, average anxiety, and low anxiety. The study's findings showed that most teachers (as learners) participants experienced high and average anxiety levels. However, research shows that the anxiety level among the participants decreased with time and training. As the subjects in the current study have only trained for one semester, there are positive chances for participants to experience less anxiety until the end of the course. Many researchers conducted studies to explore writing anxiety among university EFL participants and found them to feel high and average levels of anxiety (Hassan, 2001; Latif, 2007; Huwari and Abd Aziz, 2011; Al-Sawalha and Chow, 2012). The second research question about the role of gender in determining learners' anxiety levels displayed no significant effect, meaning learners' anxiety levels has no connection to whether learners are male or female. However, it can be seen that male participants were relatively more anxious than females, with no significant value. Rodriguez et al. (2009) research study has displayed female participants experiencing more anxious feelings about writing than male participants. Whereas Rodriguez et al. (2009) study has reported significant effects for gender, pointing to the females' significantly higher levels of general foreign language anxiety and writing anxiety.

Similarly, Cheng (2002) claimed that gender creates differences in skill-specific foreign language anxiety. In this sense, the present study added to the inconclusive nature of gender issues. There is much research evidence where researchers found no significant difference gender-wise (Shawish and Atea, 2010; Shang, 2013).

After analyzing the inventory SLWAI according to anxiety levels and gender influence, another area to analyze was the type of anxiety. The inventory was divided into three anxiety types: cognitive, somatic, and avoidance. It was found that participants experienced cognitive anxiety more than somatic and avoidance. The third research question is the essential part of the research to determine why learners feel anxious about writing skills. Generally, anxiety is a feeling meant to be experienced by foreign language learners initially, but there are specific reasons those may enhance anxiety while writing classes and writing-related activities. To the data collected through interview questions, unlike in previous research work, most learners did not agree with the statements that showed the negative role of teachers in writing classes. Many research studies (Cheng, 2004; Atay and Kurt, 2006) displayed the negative influence of writing instructors on learners' approach to L2 writing. The statements regarding writing course books also displayed mixed ideas but no significant evidence of learners' dissatisfaction. The reasons for learners experiencing writing anxiety seemed to connect more to their writing ability and writing knowledge. The majority of learners shared a lack of vocabulary and appropriate linguistic expressions. One of the frequently felt writing anxiety reasons is grammatical accuracy, which most learners feel lacking. Command over lexical resources and grammatical range proved essential factors that make learners anxious while writing in a foreign language. The other causes determined that lead to anxiety in teachers (as learners) toward L2 writing offer an additional contribution to the previous research, including linguistic difficulties such as inadequate vocabulary and grammar knowledge (Gkonou, 2011), insufficient past writing practices (Atay and Kurt, 2006), fear of negative evaluation from the peers (Chang, 2004; Maria, 2006), lack of generating and organizing ideas (Alnufaie and Grenfell, 2013), lack of self-confidence (Latif, 2007; Aljafen, 2013), lack of topical knowledge or uninterested topic (Lee et al., 2001), and time constraints (Chang, 2004). Additionally, the inventory findings indicated that the course book might negatively influence anxiety if the content does not contain suitable explanations and examples to teach writing.

## Conclusion

According to statistical and qualitative research, most pre-service teachers (as learners) exhibit high or average anxiety. Learners' writing anxiety was found to be unrelated to their gender. Moreover, different factors arose, ranging from linguistic challenges and fear of negative judgment to a lack of self-confidence and bad prior experiences. Unlike many other studies, participants in this one did not blame their nervous feelings on their teachers' instructional strategies or feedback preferences. Given the widespread perception that L2 writing anxiety is an under-researched topic, this study could help increase our awareness of the numerous dimensions of second language writing anxiety and encourage much scholarly work to look into the matter from other angles. Nonetheless, the study may fail to produce generalizability of results by keeping various constraints, having a small number of participants, and involving non-native pre-service English teachers as participants. A suggested idea for future research is to undertake such anxiety studies with a more significant number of participants to obtain more reliable results.

### Future implications

The findings of this study could have significant ramifications for language and teacher education programs. Instructors should know that worry harms learners' writing in their second language, even if they are experienced EFL teachers (learners in the present case). Instructors should also be aware of this detrimental effect before attributing learners' inability to write to a lack of enthusiasm, skills, or boredom with the lesson. Some anxiety-relieving activities may aid learners in overcoming the unpleasant emotions that they bring to the foreign language lesson. Therefore, some teacher training programs or seminars on how to motivate their learners to write and how to react to their written products in terms of choosing the proper error correction strategy and organizing the class so that other learners do not comment or laugh at someone's mistake can be arranged. Furthermore, teachers may provide some intriguing and current themes to the class to encourage learners to write, or they may use topics with which the learners are already familiar (Rankin-Brown and Fitzpatrick, 2007). Peer feedback (Grabe and Kaplan, 1996), ungraded writing tasks such as journal writing on a topic (Clark, 2005), and teaching vocabulary-expansion tools may also aid in resolving the issue. Discussions before writing tasks on learners' compositions may be linked to worry, but they also facilitate writing by providing a more secure ground to focus. Most crucially, the findings call for rethinking how much time, and information language learners are exposed to when writing. Suppose the goal is to educate and enhance writing skills. Training should begin early in the language learning process, even in elementary or secondary schools, using a process-based approach, as many studies have highlighted the anxiety-inducing influence of those who use product-based pedagogies. More research into techniques to decrease writing anxiety appears to be of the utmost importance.

## Author contributions

UR: idea development, theoretical framework, and analysis. JQ: project supervisor. MA: theoretical framework and method. All authors contributed to the article and approved the submitted version.
